# Comparison of TG‐43 dosimetric parameters of brachytherapy sources obtained by three different versions of MCNP codes

**DOI:** 10.1120/jacmp.v17i2.5797

**Published:** 2016-03-08

**Authors:** Neda Zaker, Mehdi Zehtabian, Sedigheh Sina, Craig Koontz, Ali S. Meigooni1

**Affiliations:** ^1^ Radiation Medicine Engineering Department School of Mechanical Engineering Shiraz University Shiraz Fars Iran; ^2^ Radiation Research Center School of Mechanical Engineering Shiraz University Shiraz Fars Iran; ^3^ Comprehensive Cancer Centers of Nevada Las Vegas NV USA; ^4^ School of Allied Health Sciences University of Nevada Las Vegas Las Vegas NV USA

**Keywords:** brachytherapy, MCNP, dosimetry, TG‐43

## Abstract

Monte Carlo simulations are widely used for calculation of the dosimetric parameters of brachytherapy sources. MCNP4C2, MCNP5, MCNPX, EGS4, EGSnrc, PTRAN, and GEANT4 are among the most commonly used codes in this field. Each of these codes utilizes a cross‐sectional library for the purpose of simulating different elements and materials with complex chemical compositions. The accuracies of the final outcomes of these simulations are very sensitive to the accuracies of the cross‐sectional libraries. Several investigators have shown that inaccuracies of some of the cross section files have led to errors in ${\;}^{125}I$ and ${\;}^{103}\text{Pd}$ parameters. The purpose of this study is to compare the dosimetric parameters of sample brachytherapy sources, calculated with three different versions of the MCNP code — MCNP4C, MCNP5, and MCNPX. In these simulations for each source type, the source and phantom geometries, as well as the number of the photons, were kept identical, thus eliminating the possible uncertainties. The results of these investigations indicate that for low‐energy sources such as ${\;}^{125}I$ and ${\;}^{103}\text{Pd}$ there are discrepancies in $g_{L}(r)$ values. Discrepancies up to 21.7% and 28% are observed between MCNP4C and other codes at a distance of 6 cm for ${\;}^{103}\text{Pd}$ and 10 cm for ${\;}^{125}I$ from the source, respectively. However, for higher energy sources, the discrepancies in $g_{L}(r)$ values are less than 1.1% for ${\;}^{192}\text{Ir}$ and less than 1.2% for ${\;}^{137}\text{Cs}$ between the three codes.

PACS number(s): 87.56.bg

## I. INTRODUCTION

Brachytherapy has been a cornerstone treatment technique for the management of various malignancies such as gynecological, prostate, and breast tumors. In low‐dose‐rate (LDR) brachytherapy treatments, ${\;}^{125}I,{\;}^{125}\text{Pd},{\;}^{125}\text{Ir}$, and ${\;}^{137}\text{Cs}$ sources have been utilized for many years. Task Group 43 (TG‐43) of the American Associations of Physicists in Medicine (AAPM)^1^, ^2^ has recommended the dosimetric evaluation of low energy brachytherapy sources using experimental technique and/or Monte Carlo simulations.^3^, ^4^, ^5^, ^6^, ^7^, ^8^, ^9^, ^10^, ^11^, ^12^, ^13^, ^14^, ^15^ A subsequent Working Group from AAPM has recommended the dosimetric evaluation of high‐energy sources^(16)^ using a similar method. Several different Monte Carlo codes have been utilized for the dosimetric evaluation of brachytherapy sources including MCNP4C2,^17^, ^18^ MCNP5,^(19)^ MCNPX,^(20)^ EGS4,^(21)^ EGSnrc,^(22)^ PTRAN,^(5)^ and GEANT4.^(23)^ Each of these codes uses a different cross‐sectional library for both photons and electrons for all the chemical elements within the energy range of 1 eV to 1 GeV. The accuracies of the simulated data have been demonstrated by comparison with the experimental data.^9^, ^11^ However, the large statistical uncertainties of the experimental data masked the true uncertainties of the Monte Carlo simulations due to the cross‐sectional errors or selection of insufficient number of photons in the simulations. In 2002, DeMarco et al.^(7)^ performed an extensive study regarding the accuracies of cross‐sectional data utilized in different Monte Carlo simulation codes and their impacts on the absorbed doses and collisional kerma values. They reported up to 10% differences in the photoelectric cross sections for water at 30 keV between the DLC‐200 from RSICC at Oak Ridge National Laboratory and XCOM cross‐sectional database maintained at the National Institute of Standards and Technology.^24^, ^25^ Furthermore, they demonstrated that differences of up to 10% are observed in the photoelectric cross section for water at 30 keV between the standard MCNP cross‐sectional dataset (DLC‐200) and the most recent XCOM/NIST tabulation. More specifically, they had shown that at energies of 20 keV and 30 keV, the absolute dose rates in water at 1.0 cm depth increased by 3.5% and 7.1%, respectively, when the cross‐sectional tabulated data were changed from DLC‐200 to XCOM/NIST data. However, these changes were found to be insignificant when using DLC‐146.^(26)^

The exact cause of the differences between various Monte Carlo reported data generated by different investigators for the same source type was left unresolved. For example, the dose rate constant of ${\;}^{125}I$, Model 6733 was reported to be $0.965\;{\text{cGyh}}^{-1}U^{-1}$ by Mosleh‐Shirazi et al.^(19)^ using the MCNP5 code, while it is has been reported to be $0.993\;{\text{cGyh}}^{-1}U^{-1}$ by the same authors using the MCNP4C2 code and $0.970\;{\text{cGyh}}^{-1}U^{-1}$ by Sowards and Meigooni^(27)^ using the PTRAN code. There is a need to understand whether the differences between these values are due to the errors in the cross‐sectional data which has been used or some other cause. These causes could include the differences in the geometrical designs of the sources and phantoms used in each project, the differences in number of histories used for simulations, or size of the tally cells. This project is designed to resolve these issues.

The aim of this project is to verify the differences among three versions of the MCNP Monte Carlo codes (MCNP4C2, MCNP5, and MCNPX), which are widely utilized for dosimetry of brachytherapy sources. The TG‐43 parameters of four different brachytherapy sources — ${\;}^{192}\text{Ir}$ (model Vari‐Source VS2000), ${\;}^{125}I$ (model IAI‐125A), ${\;}^{103}\text{Pd}$ (model Best 2335) and ${\;}^{137}\text{Cs}$ (Selectron pellet sources from Nucletron) — have been determined using these codes. In the simulations for each source type, the phantom and source geometries, the number of particle histories/photons, the size of the tally cells, and dose points were kept identical for all the Monte Carlo codes. The cross‐sectional files used in the MCNP5, MCNP4C2, and MCNPX are ENDF/B‐VI release 8,^(28)^ ENDF/B‐VI version 5,^(29)^ and ENDF/B‐VI release 8,^(28)^ respectively.

## II. MATERIALS AND METHODS

### A. Source characteristics

Four different commercially available brachytherapy sources were selected for this project. These sources are the Varian VariSource ${\;}^{192}\text{Ir}$, Model VS2000 (Varian, Varian Medical Systems, Palo Alto, CA), IsoAid Advantage ${\;}^{125}I$ Model IAI‐125A (IsoAid LLC, Port Richey, FL), the Best Industries ${\;}^{103}\text{Pd}$ Model Best 2335 (Best International, Best Medical International, Inc., Springfield, VA), and Nucletron ${\;}^{137}\text{Cs}$ Selectron pellet sources (Nucletron BV, Veenedaal, The Netherlands). These sources were selected as representative of each source type and the choices do not reflect endorsement of the vendors. The physical characteristics of these sources are briefly described below.

#### A.1 ${\;}^{125}I$

**Figure 1:**
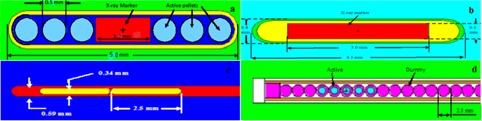
The Schematic diagram of the simulated sources: (a) the Best Industries ${\;}^{103}\text{Pd}$ Model Best 2335, (b) IsoAid Advantage ${\;}^{125}I$ Model IAI‐125A, (c) the Varian VariSource ${\;}^{192}\text{Ir}$, and (d) Nucletron ${\;}^{137}\text{Cs}$ Selectron pellet sources.

The IsoAid Advantage ${\;}^{125}I$ (model IAI‐125A) source was introduced in the North American market in 2002.^(9)^ Dimensions of this source for the Monte Carlo simulations are taken from the study by Meigooni et al.^(9)^ The IsoAid Advantage seed contains a 3 mm long silver rod X‐ray marker with a diameter of 0.50 mm. The silver rod is coated with a 1 μm thick layer of AgI containing ${\;}^{125}I$. The active silver rod is encapsulated in 0.05 mm thick titanium (Ti) capsule with an outside diameter of 0.80 mm. The laser‐welded end‐caps have a maximum thickness of 0.10 mm. Two hemispheres are used for modeling the end‐caps, one with 0.40 mm radius Ti hemisphere overlapped with a 0.35 mm radius air. The center of the air hemisphere was shifted by 0.05 mm relative to the Ti hemisphere. The overall source length is 4.50 mm and the active length is 3.0 mm. The cylindrical source element is free to move approximately 0.150 mm along the longitudinal direction of the source and 0.100 mm in the radial direction. The schematic diagram of the simulated source is shown in Fig. 1(a).

#### A.2 ${\;}^{103}Pd$

The ${\;}^{103}\text{Pd}$ source (Model Best 2335) was obtained from Best Industries. The dimensions of the source were taken from previous investigations.^(11)^ The Best 2335 source consists of a cylindrical tungsten X‐ray marker with a diameter of 0.5 mm and a length of 1.2 mm. There are three polymer spheres with 0.5 mm diameter located on either side of the X‐ray marker. The centers of these pellets are located at distances of 0.9 mm, 1.5 mm, and 2.1 mm relative to the center of the seed. The relative chemical compositions of these spheres by weight are: 89.73% C, 7.85% H, 1.68% O, and 0.740% N. In addition, the mass density of the polymer is assumed to be 1.00 g/cm^3^. The polymer spheres are uniformly coated in ${\;}^{103}\text{Pd}$. The spheres and the X‐ray marker are double encapsulated in titanium shell with total thickness of 0.080 mm and outer diameter of 0.800 mm. The overall source length is 5.00 mm and the active length is 4.76 mm. The maximum possible displacement of a source sphere is 0.570 mm along the seed axis and up to 0.070 mm in the radial direction. The schematic diagram of the simulated source is shown in Fig. 1(b).

#### A.3 ${\;}^{192}Ir$

The source geometry and dimensions of the VariSource VS2000 were taken from the investigation of Angelopoulos et al.,^(13)^ Papagiannis et al.,^(14)^ and Llisp et al.^(15)^ The VS2000 consists of two 2.50 mm long sources pellets. Each source pellet is made up of a 2.16 mm long cylindrical section with a 0.34 mm diameter and hemispherical ends with the same diameter. The two sources are placed laser drilled hole made at the end of a solid Ni/Ti wire (55.6%/44.4%) with a diameter of 0.59 mm. The end of the wire was sealed with a 1 mm thick plaque. This source end was modeled as a hemisphere with its center shifted 3.205 mm relative to the center of the source. During the simulation, the length of the wire was extended by about 5.0 cm from the center of the source to include the impact of the source wire on the dose distribution. The active length of this source is 5.0 mm. The schematic diagram of the simulated source is shown in Fig. 1(c).

#### A.4 ${\;}^{137}Cs$

Spherical ${\;}^{137}\text{Cs}$ pellet sources of the LDR Selectron remote afterloading system, with 2.50 mm in diameter encapsulated in 0.5 mm stainless steel material, was simulated for this source.^(8)^ The active cores of the sources contain spherical borosilicate glass with 1.50 mm diameter. For treatment of gynecological diseases, different combinations of active pellet sources and dummy pellets are moved through a plastic catheter and stopped at the end of the applicator by a stainless stopping screw.

In this study, a source train containing five active Amersham pellets was considered at the end of the cylindrical applicator to simulate a linear ${\;}^{137}\text{Cs}$ source, with 1 cm active length. The schematic diagram of the simulated source is shown in Fig. 1(d).

### B. TG‐43 dose calculations

The AAPM Task Group 43 (TG‐43^)(1‐2)^ introduced a formalism for calculation of dose distribution around a low‐energy sealed brachytherapy source. Also, there is a report from the AAPM and ESTRO for dose calculation around photon‐emitting brachytherapy sources with average energy higher than 50 keV.^(16)^ We have used this report for dosimetric evaluation of ${\;}^{137}\text{Cs}$ and ${\;}^{192}\text{Ir}$. This new report explains the differences of low‐energy sources and high‐energy sources for distances very close to the sources. At larger distances, there are not significant differences between the dosimetry of the sources using the two recommendations. Since the goal of this project is toward the dosimetry of the source for distances greater than 1 cm, the updated TG‐43 protocol^(2)^ (i.e., TG‐43U1) will be used for all the sources.

According to the TG‐43U1 formalism,^(2)^ the dose rates around brachytherapy sources are obtained from:

(1) $$\dot{D}=S_{K\cdot \Lambda \cdot }\frac{G_{l}(r,\theta )}{G_{l}(r_{0},\theta_{0})}g_{L}(r)F(r,\theta )$$

where $S_{K}$ is the air‐kerma strength of the source, Λ is the dose rate constant, $G_{L}(r,\theta )$ is the geometry function, $g_{L}(r)$ is the radial dose function, and F(r,θ) is the 2D anisotropy function. The $\left(r_{0},\theta_{0}\right)$ is the reference point relative to the source with $r_{0}=1\;\text{cm}$ and $\theta_{0}=\pi /2$. The subscript “L” has been added in TG‐43U1^(2)^ to denote the linear source approximation. The air‐kerma strength, $S_{K}$ is the air‐kerma rate ${\dot{K}}_{\delta }\;(d)$ multiplied by the square of the distance for energy levels greater than δ:

(2) $$S_{K}={\dot{K}}_{\delta }(d).d^{2}$$

Δ is the ratio of the dose rate at the reference point, $P(r_{0},\theta_{0})$ and $S_{K}$. The unit of is ${\text{cGyh}}^{-1}\;U^{-1}$:

(1) $$\dot{D}=S_{K\cdot \Lambda \cdot }\frac{G_{l}(r,\theta )}{G_{l}(r_{0},\theta_{0})}g_{L}(r)F(r,\theta )$$

The geometry function accounts for the activity distribution within the source and the distance between the point of interest and the source. TG‐43U1 defines the geometry function as

(4) $$\begin{array}{l} G_{L}(r,\theta )=\{\begin{array}{c} \frac{\beta }{Lr\text{sin}\theta }\;if\theta \neq 0\\ {\left(r^{2}-\frac{L^{2}}{4}\right)}^{-1}\;if\theta =0 \end{array}\\ G_{L}(r)=\frac{\dot{D}(r,\frac{\pi }{2})G_{L}(r_{0,}\frac{\pi }{2})}{\dot{D}(r_{0},\frac{\pi }{2})G_{L}(r_{,}\frac{\pi }{2})} \end{array}$$

where β is the angle in radians, subtended by the point of interest, P(r,θ), to the tips of the active length of a hypothetical line; *L* is the active length of the source. The radial dose function, $g_{L}(r)$, takes into account the effects of photon scattering and attenuation and is defined as follows:

(5) $$G_{L}(r)=\frac{\dot{D}(r,\frac{\pi }{2})G_{L}(r_{0,}\frac{\pi }{2})}{\dot{D}(r_{0},\frac{\pi }{2})G_{L}(r_{,}\frac{\pi }{2})}$$

The 2D anisotropy function describes the variations in dose as a function of polar angle relative to the transverse plane and is defined as:

(6) $$F(r,\theta )=\frac{\dot{D}(r,\theta )}{\dot{D}(r,\theta_{o})}\frac{G(r,\theta_{o})}{G(r,\theta )}$$

### C. MCNP code

MCNP is a general purpose Monte Carlo radiation transport code that can be used for neutron, photon, electron, or coupled neutron/photon/electron transport.^(30)^ Three versions of the MCNP code have been used in this study: MCNP4C2, MCNP5, and MCNPX. When a version of MCNP has been released, newly introduced features may be based on those from previous versions or these features may be novel to MCNP. However, the sources of these new features are not always explicit.

#### C.1 MCNP4C2

MCNP4C2 is the first major release of MCNP since version MCNP4B (February 4, 1997).^(31)^ One of the major new features of MCNP4C2 relative to version MCNP4B is the use of ENDF/B‐VI improvements. Monte Carlo simulations are, in general, only as accurate as the underlying cross‐sectional libraries.^(31)^ Several investigators^7^, ^32^ have discussed the problems with the photon cross‐sectional data that were included in MCNP^(31)^ simulations. The effect of these problems is especially visible in dose calculations involving low‐energy photon emitting seeds. In 2002, the photon cross‐sectional data included with the standard distribution of MCNP was completely revised (X‐5 Monte Carlo Team, 2003).^(31)^

For MCNP4C2 there are two photon interaction data libraries Labeled as ZZZ000.nnP with nn=01 and nn=02.^(29)^ For the ZAID = ZZZ000.01P library, the photon interaction tables were created for elements from z=1 through z=94 except z=84, 85, 87, 88, 89, 91, and 93. ZAID is Nuclide Identification number. This number is used to identify the element or nuclide desired. The form of the number is ZZZAAA.nnX, where ZZZ is the atomic number of the element or nuclide, AAA is the mass. These tables were based on evaluated data from ENDF^(33)^ from 1 keV to 100 MeV. However, in the ZAID=ZZZ000.02P library, the values were a subset of the ZAID=ZZZ000.01P library with pair production thresholds added for the Storm‐Israel^(34)^ data. Data above 15 MeV for the Storm‐Israel data and above 100 MeV for the ENDF data come from adaptation of the Livermore Evaluated Photon data library (EPDL^(35)^) and are valid for energies up to 100 GeV.^(29)^ For this project we used data library for nn=02.^(34)^

#### C.2 MCNP5

The MCNP5 code incorporates an arbitrary three‐dimensional configuration for materials in geometric cells bounded by first‐ and second‐degree surfaces and fourth‐degree elliptical tori. For photons, the code accounts for incoherent and coherent scattering, the possibility of fluorescent emission after photoelectric absorption, and the absorption in electron‐positron pair‐production.^(28)^ There are four photon transport libraries maintained by X‐5 and distributed with MCNP: MCPLIB, MCPLIB02, MCPLIB03, and MCPLIB04.^(35)^ MCPLIB04 was officially released in 2002.^(29)^ The cross section, form factor, and fluorescence data are all derived from the ENDF/B‐VI.8 data library that are derived from EPDL97.^(34)^ Cross‐sectional data are given for incident photon energies from 1 keV to 100 GeV. Fluorescence data are derived from the atomic relaxation data available in ENDF/B‐VI.8, but use the storage and sampling scheme defined by Everett and Cashwell.^(33)^

Electron interaction data tables are required both for problems in which electrons are actually transported, and for photon problems in which the thick‐target bremsstrahlung model is used. Electron data tables are identified by ZAIDs of the form ZZZ000.nnE, and are selected by default when the problem mode requires them. There are two electron interaction data libraries: el01 (ZAID endings of .01e) and el03 (ZAID endings of .03e).^(30)^ The electron libraries contain data on an element‐by‐element basis for atomic numbers from Z equal 1 to 94. The library data contain energies for tabulation, radiative stopping power parameters, bremsstrahlung production cross sections bremsstrahlung energy distributions, K‐edge energies, and Auger electron production energies. It also contains parameters for the evaluation of the Goudsmit‐Saunderson theory^(29)^ for angular deflection based on the Riley cross‐sectional calculation. This file includes Mott correction factors to the Rutherford cross sections used in the Goudsmit‐Saunderson theory. The el03 library includes the atomic data of Carlson that was used in the density effect calculation.^(30)^

#### C.3 MCNPX

MCNPX is a general purpose Monte Carlo radiation transport code that tracks nearly all particles, as well as photons and electrons, at almost all energies. It is a superset of MCNP4C3^(36)^ and has many capabilities beyond MCNP4C3 such as heavy ion transport and long file names. It was first released to the public in 1999 as version 2.1.5. Many new tally sources and variance reduction options have been developed and new tallies have been created specific to the intermediate and high‐energy physics ranges.^(36)^ The MCNPX photon transport libraries are identical to the MCNP5 libraries that were defined in the previous section. The major difference of MCNP5 and MCNPX is their energy ranges. The energy ranges for MCNP5 are 1 keV to 1 GeV for electrons, and 1 keV to 100 GeV for photons.

#### C.4 Revised MCNP4C

Another code that has been generated in this project (referred here after as MCNP4C‐revised) is the original MCNP4C2 code with the cross‐sectional library taken from the MCNP5 and MCNPX library. Therefore, the cross‐sectional library is changed from ENDF/B‐VI version 5 to ENDF/B‐VI. 8. This adjustment is made in order to identify if the differences between MCNP4C2 and other codes are simply the differences in their cross‐sectional library or if changes in the code are responsible for these differences.

### D. Phantom geometry

To obtain the dose rate constant, Δ, radial dose function, $g_{L}(r)$, and the anisotropy function, F(r,θ), the simulations were performed in a spherical water phantom with 30 cm radius. Each source type was placed at the center of the phantom, and spherical tally cells were defined at different distances from the source center. The radius of the tally cells was 0.02 cm for distances less than 2 cm and 0.05 cm for distances greater than 3 cm. For each simulation, 10^9^ particle histories were considered to ensure that the relative errors ($R(\%)=100*\frac{S_{\bar{x}}}{\bar{X}}$ that represents the estimated relative error (%) at the 1 σ level) of MCNP simulations at all distances are less than 1%. To obtain air‐kerma strength, each source type was placed at the center of an air phantom, the kerma rates were obtained in spherical tally cells, using F6‐tally. The air‐kerma rates, obtained for distances 1 to 10 cm, were multiplied by the square of the distance to obtain the air‐kerma strength. The average values of the air‐kerma strength were considered as the $S_{K}$ value for each source. In all simulations *F4 and F6 tallies were used. The results of the

*F4 tally were multiplied by the values of mass absorption coefficients, to convert them to dose values. The TG‐43 parameters (i.e., dose rate constant, $g_{L}(r)$, and F(r,θ)) were obtained according to Eq. (2) to (6). The uncertainty analysis of each parameter was obtained using the error propagation method. F(r,θ) is obtained at distances from 1 cm to 10 cm and the angles of 0° to 180° by 10° spacing. Radial dose functions have been obtained at distances of 1 cm to 6 cm of the source. The photon energy spectrum for ${\;}^{125}I$ and ${\;}^{103}\text{Pd}$ were taken from the TG43U1 report,^(2)^ and the spectrum for ${\;}^{192}\text{Ir}$ was taken from national nuclear data center.^(37)^ For ${\;}^{137}\text{Cs}$, 662 keV monoenergetic gamma rays were considered.

## III. RESULTS

### A. Dose rate constant

Table 1 shows the dose rate constant of ${\;}^{103}\text{Pd},{\;}^{125}I,{\;}^{192}\text{Ir},{\;}^{137}\text{Cs}$ sources obtained by the four different MCNP codes and also the published values by different investigators.^9^, ^11^, ^13^, ^38^, ^39^ For a better visibility of the differences between different Monte Carlo codes, these results were compared with the values obtained from the MCNP5 code (the most recently used code). Differences between the data from all the codes are excellent (0.1%) except MCNP4C2 that is 4.4% and 1.1% for ${\;}^{103}\text{Pd}$ and ${\;}^{125}I$, respectively. It should be noted that the results of the simulations by the MCNP4C‐revised code was similar to those of MCNP5 and MCNPX codes. Therefore, the main source of error for the dose rate constant of the ${\;}^{103}\text{Pd}$ and ${\;}^{125}I$ from MCNP4C2 code was the use of the cross‐sectional file.

The uncertainty of dose rate constant, originates from two parameters: $S_{K}$ and $\dot{D}\cdot (r_{0},\theta_{0})$. The error of $S_{K}$ is calculated by the quadratic summation of the uncertainties from a point dose and the values calculated by deviations from the fitted line source. The uncertainty of $\dot{D}\cdot (r_{0},\theta_{0})$ is calculated statistical fluctuations by the MCNP codes. Then, the uncertainty of the dose rate constant is calculated following the error propagation formula.^(32)^

**Table 1:** The dose rate constant $\left(\Delta :{\text{cGyh}}_{-1}U^{-1}\right)$ of ${\;}^{103}\text{Pd},{\;}^{125}I,{\;}^{192}\text{Ir}$, and ${\;}^{137}\text{Cs}$ sources

*Source*	*Method*	*Dose Rate Constant* $\left({\text{cGy}}^{-1}U^{-1}\right)$	*Differences Relative to the MCNP5 Value*
${\;}^{103}\text{Pd}$	MCNP4C2	0.726±0.6%	4.6%
	MCNPX	0.695±0.6%	0.1%
	MCNP5	0.694±0.6%	
	MCNP4C‐Revised	0.695±0.6%	0.1%
	Meigooni et al.^(11)^	0.69	−0.6%
	TG‐43U1S1^(38)^	0.685	−1.3%
	Taylor & Rogers^(39)^	0.650	−6.3%
${\;}^{125}I$	MCNP4C2	0.935±0.6%	1.1%
	MCNPX	0.924±0.6%	−0.1%
	MCNP5	0.925±0.6%	
	MCNP4C‐Revised	0.928±0.6%	0.3%
	Meigooni et al^(9)^	0.99	7%
	TG‐43U1S1^(38)^	0.981	6%
	Taylor & Rogers^(39)^	0.925	0.0%
${\;}^{192}\text{Ir}$	MCNP4C2	1.119±0.5%	0.1%
	MCNPX	1.119±0.5%	0.1%
	MCNP5	1.118±0.5%	
	MCNP4C‐Revised	1.119±0.5%	0.1%
	Angelopoulos et al.^(13)^	1.101	−1.5%
	Taylor & Rogers^(22)^	1.099	−1.7%
${\;}^{137}\text{Cs}$	MCNP4C2	0.993±0.5%	0.2%
	MCNPX	0.995±0.5%	0.0%
	MCNP5	0.995±0.5%	0.0%
	MCNP4C‐Revised	1.007±0.5%	1.2%

### B. Radial dose function

**Figure 2:**
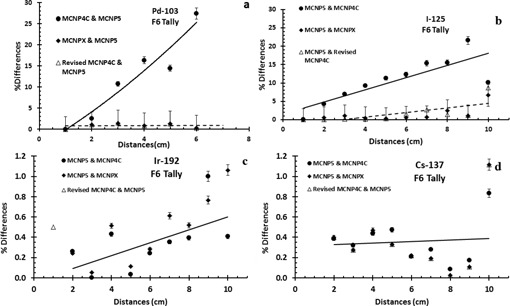
The percentage differences between the radial dose functions of ${\;}^{103}\text{Pd}$ (a), ${\;}^{125}I$ (b), ${\;}^{192}\text{Ir}$ (c), and ${\;}^{137}\text{Cs}$ (d) sources calculated by different MCNP codes and the values obtained by the MCNP5 code using the F6 tally.

Table 2 shows the simulated radial dose functions of the ${\;}^{103}\text{Pd},{\;}^{125}I,{\;}^{192}\text{Ir},{\;}^{137}\text{Cs}$ sources as a function of radial distance, obtained by the four different Monte Carlo codes, using *F4 and F6 tally options. The percentage difference between the results of these codes relative to the MCNP5 data sets for ${\;}^{103}\text{Pd}$ and ${\;}^{192}\text{Ir}$ are shown in Fig. 2. These results indicate that for the F6 tally option, the percentage differences between the values obtained by the MCNP5 and MCNPX Monte Carlo codes are less than 6.7% for all distances. However, the percentage differences between the data obtained by the MCNP4C2 and MCNP5 codes increases with increasing distance from the source. The percentage differences between MCNP4C2 and MCNP5 codes at 6 cm distance from the ${\;}^{103}\text{Pd}$ source reach approximately 27% and 28% for F6 and *F4 tallies, respectively. However, the percentage differences between the MCNP4C2 and MCNP5 for ${\;}^{125}I,{\;}^{192}\text{Ir}$, and ${\;}^{137}\text{Cs}$ sources at a distance of 10 cm are less than 10.5%, 0.5%, and 1%, respectively. Interestingly, the results of the simulations by the MCNP4C‐revised code are similar to those of MCNP5 and MCNPX codes. Therefore, the main source of error for the radial dose function of the low‐energy sources from MCNP4C2 code was its cross‐sectional

**Table 2:** Comparison of the radial dose function of ${\;}^{103}\text{Pd}$ source obtained by F6 and *F4 tally options

*Source*	*r (cm)*	MCNP4C2 *F4 tally	*MCNP4C2 F6 tally*	MCNP5 *F4 tally	*MCNP5 F6 tally*	MCNPX *F4 tally	*MCNPX F6 tally*	MCNP4C‐Revised *F4 tally	*MCNP4C‐Revised F6 tally*
${\;}^{103}\text{Pd}$	1	1.00E+00 1.00E+00	1.00E+00 1.00E+00	1.00E+00 1.00E+00	1.00E+00 1.00E+00	1.00E+00 1.00E+00	1.00E+00 1.00E+00	1.00E+00 1.00E+00	1.00E+00 1.00E+00
	2	6.04E−01	6.03E−01	5.89E−01	5.89E−01	5.83E−01	5.82E−01	5.82E−01	5.82E−01
	3	3.52E−01	3.52E−01	3.18E−01	3.18E−01	3.23E−01	3.23E−01	3.23E−01	3.22E−01
	4	1.96E−01	1.95E−01	1.68E−01	1.68E−01	1.69E−01	1.69E−01	1.69E−01	1.69E−01
	5	1.12E−01	1.11E−01	9.73E−02	9.73E−02	9.61E−02	9.61E−02	9.61E−02	9.61E−02
	6	6.92E−02	6.90E−02	5.42E−02	5.42E−02	5.41E−02	5.41E−02	5.41E−02	5.41E−02
${\;}^{125}I$	1	1.0000	1.0000	1.0000	1.0000	1.0000	1.0000	1.0000	1.0000
	2	0.8483	0.8484	0.8165	0.8137	0.8180	0.8159	0.8171	0.8143
	3	0.6797	0.6801	0.6358	0.6357	0.6422	0.6385	0.6368	0.6368
	4	0.5369	0.5372	0.4913	0.4913	0.4936	0.4907	0.4904	0.4904
	5	0.4141	0.4146	0.3721	0.3722	0.3731	0.3709	0.3711	0.3710
	6	0.3119	0.3121	0.2779	0.2778	0.2762	0.2746	0.2735	0.2735
	7	0.2402	0.2400	0.2081	0.2080	0.2053	0.2065	0.2024	0.2023
	8	0.1752	0.1750	0.1519	0.1516	0.1544	0.1553	0.1539	0.1538
	9	0.1365	0.1365	0.1123	0.1123	0.1105	0.1111	0.1110	0.1109
	10	0.0961	0.0961	0.0873	0.0873	0.0810	0.0814	0.0796	0.0796
${\;}^{192}\text{Ir}$	1	1.0000	1.0000	1.0000	1.0000	1.0000	1.0000	1.0000	1.0000
	2	0.9932	0.9933	0.9958	0.9958	0.9934	0.9936	0.9935	0.9936
	3	1.0036	1.0049	1.0040	1.0036	1.0031	1.0038	1.0042	1.0036
	4	0.9990	1.0008	1.0044	1.0033	0.9981	0.9987	0.9997	0.9990
	5	0.9986	0.9960	1.0018	0.9989	1.0001	0.9948	0.9950	0.9999
	6	0.9928	0.9971	0.9965	0.9952	0.9923	0.9939	0.9938	0.9926
	7	0.9729	0.9681	0.9732	0.9715	0.9672	0.9656	0.9675	0.9664
	8	0.9555	0.9432	0.9459	0.9395	0.9524	0.9444	0.9446	0.9426
	9	0.9338	0.9313	0.9391	0.9407	0.9320	0.9335	0.9340	0.9321
	10	0.9243	0.9243	0.9122	0.9141	0.9277	0.9238	0.9265	0.9240
${\;}^{137}\text{Cs}$	1	1.0000	1.0000	1.0000	1.0000	1.0000	1.0000	1.0000	1.0000
	2	0.9909	0.9938	0.9978	0.9948	0.9908	0.9938	0.9938	0.9908
	3	0.9883	0.9942	0.9909	0.9852	0.9879	0.9941	0.9941	0.9879
	4	0.9853	0.9935	0.9891	0.9810	0.9856	0.9939	0.9939	0.9856
	5	0.9706	0.9805	0.9763	0.9661	0.9693	0.9794	0.9794	0.9693
	6	0.9401	0.9527	0.9538	0.9421	0.9400	0.9526	0.9526	0.9400
	7	0.9526	0.9386	0.9496	0.9360	0.9520	0.9378	0.9520	0.9378
	8	0.9234	0.9053	0.9188	0.9045	0.9227	0.9047	0.9196	0.9047
	9	0.8838	0.8680	0.8840	0.8695	0.8842	0.8686	0.8835	0.8686
	10	0.8852	0.8540	0.9000	0.8612	0.8850	0.8516	0.8680	0.8516

file. A comparison of the simulated radial dose function of the sources in this project using F6 tally with the published data is shown in Fig. 3.

**Figure 3:**
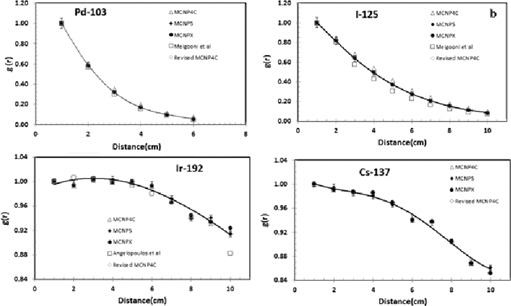
Comparison of the Monte Carlo simulated radial dose functions of ${\;}^{103}\text{Pd}$ (a), ${\;}^{125}I$ (b), ${\;}^{192}\text{Ir}$ (c), and ${\;}^{137}\text{Cs}$ (d) sources obtained by the four MCNP codes in this project, using F6 tally. These results are also compared with the published data by Meigooni et al.^(11)^ for ${\;}^{103}\text{Pd}$, Meigooni et al.^(9)^ for ${\;}^{125}I$, and Angelopoulos et al.^(13)^ for ${\;}^{192}\text{Ir}$.

### C. Anisotropy function

**Figure 4:**
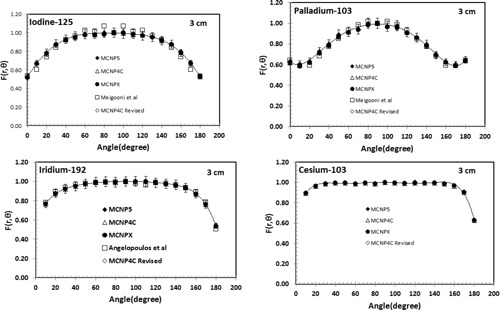
Comparison of the Monte Carlo simulated 2D anisotropy functions of ${\;}^{125}I$ (a), ${\;}^{103}\text{Pd}$ (b), ${\;}^{192}\text{Ir}$ (c), and ${\;}^{137}\text{Cs}$ (d) sources obtained by the three MCNP codes and the revised MCNP4C2 code using F6 tally. These results are also compared with the published data by Meigooni et al.^(11)^ for ${\;}^{103}\text{Pd}$, Meigooni et al.^(9)^ for ${\;}^{125}I$, and Angelopoulos et al.^(13)^ for ${\;}^{192}\text{Ir}$.

The values of 2D anisotropy function, F(r,θ), for the ${\;}^{103}\text{Pd},{\;}^{125}I,{\;}^{192}\text{Ir},{\;}^{137}\text{Cs}$ sources were obtained by MCNP4C2, MCNP5, MCNPX, and MCNP4C‐revised Monte Carlo codes at distances of 1 cm to 10 cm. Figure 4 shows the comparison of the 2D anisotropy functions of these sources by the four codes using *F4 tally and also published data by other investigators.^9^, ^11^, ^13^, ^38^ Similar results were obtained by F6 tally. No significant differences between different codes had been observed.

## IV. DISCUSSION & CONCLUSION

Four versions of Monte Carlo codes (MCNP4C2, MCNPX, MCNP5, and MCNP4C‐revised) were used to obtain the TG‐43 dosimetry parameters for low‐ and high‐energy brachytherapy sources. To demonstrate the variation of the results between the codes, all the simulated parameters were compared with the data from the MCNP5 code. The results of these investigations for the high energy brachytherapy sources (i.e., ${\;}^{192}\text{Ir}$ and ${\;}^{137}\text{Cs}$) indicated that the differences among the data from the four codes are less than 5.6%. However, in the case of low‐energy brachytherapy sources such as ${\;}^{103}\text{Pd}$ and ${\;}^{125}I$, the difference between the radial dose functions obtained by MCNP4C2 and those obtained by two other versions of the code (MCNPX and MCNP5) changes as a function of distance. The differences were found to be more than 27% for ${\;}^{103}\text{Pd}$ at r=6 cm, and more than 10% for ${\;}^{125}I$ at r=10 cm. The values of the dose rate constants for MCNP4C2 were approximately 4.4% and 1.1% larger than MCNP5 value for ${\;}^{103}\text{Pd}$ and ${\;}^{125}I$, respectively. No significant differences were found on 2D anisotropy functions.

Such differences show that the cross‐sectional library used by the MCNP4C2 code for low‐energy gamma rays were inaccurate. Therefore, the simulations were performed using the MCNP4C‐revised code, which means that the MCNP4C2 cross section was changed to that used in the MCNPX and MCNP5 (ENDF/B‐VI.8) codes. A comparison of results obtained with the MCNP4C‐revised and MCNP5 codes show that the average differences in the dose rate constant are 1.2%, 0.1%, 0.2%, and 0.1% for ${\;}^{137}\text{Cs},{\;}^{192}\text{Ir},{\;}^{125}I$, and ${\;}^{103}\text{Pd}$ respectively.

The maximum difference for *F4 and F6 tallies obtained by MCNPX and MCNP4C‐revised in all points around the source were not more than 0.01%, 0.21%, 0.67%, and 0.41% for ${\;}^{137}\text{Cs},{\;}^{192}\text{Ir},{\;}^{125}I$, and ${\;}^{103}\text{Pd}$ respectively.

The results indicate that the only difference between MCNP4C‐revised and MCNPX is the difference in their cross‐sectional libraries and, as such, the results obtained with the two codes are similar. However, the difference between the results obtained with the MCNP5 and MCNP4C‐revised codes is greater than the difference between the MCNPX and MCNP4C‐revised codes.

## ACKNOWLEDGMENTS

The authors would like to thank Ms. Michele A. Klimke, the senior dosimetrist at Comprehensive Cancer Centers of Nevada, for her valuable comments and suggestions for editorial aspects of the manuscript.

## COPYRIGHT

This work is licensed under a Creative Commons Attribution 4.0 International License.
